# August Krogh, Carbonic Acid, Combustion of Coal, and Global Warming

**DOI:** 10.1093/function/zqab052

**Published:** 2021-10-13

**Authors:** Tobias Wang

**Affiliations:** Department of Biology - Zoophysiology, Aarhus University, C.F. Møllers Allé 3, 8000 Aarhus C, Denmark

The entire globe is currently experiencing increasing temperatures and more extreme weather events caused by the continuous rise in the atmospheric levels of CO_2_ as combustion of fossil fuels, such a coal, gas, and oil, continues at an unabated rate. The rise in temperatures, the acidification of the oceans and the prolonged periods of draught represent significant problems for much of life on earth and pose an immediate challenge for almost all aspects of humanity's immediate future.

The causal influence of increased atmospheric CO_2_ on global warming is normally attributed to the Swedish physical chemist Svante Arrhenius (1859–1927), whom under the impetus of the contemporary Swedish geologist Arvid Högbom (1857–1940), was seeking an explanation for the low temperatures during the ice ages. To this end, Arrhenius performed what he later called “tedious calculations” demonstrating that atmospheric CO_2_ retains the heat emanating from the ground, and hence, also retains part of the energy that would otherwise have been lost as radiation.^1^ Being akin to the situation in a greenhouse, this mechanism was soon to be known as the “greenhouse effect.”

While much has been written about the role of Arrhenius, and how his work was based on the earlier theoretical insight from Joseph Fourier, John Tyndall, and Claude Pouillet,^2^ it is less-appreciated that the Nobel laurate in physiology August Krogh (1874–1949) contributed by extraordinarily precise measurements of atmospheric CO_2_, as well as quantitative assessment of the buffering by the oceans. The measurements were performed during an expedition to Greenland in the summer of 1902 and in his 1904 publications, Krogh points to the importance of fossils fuels in determining atmospheric CO_2_.^3,4^

August Krogh was undoubtedly one of the most influential physiologists in the early 20th century.^5,6^ He came under the tutelage of Christian Bohr at the Physiological Laboratory at Copenhagen University in 1899, and quickly embarked on studies of gas exchange in insect larvae and frogs. To this end, he developed and refined methods for very precise measurements of CO_2_ and oxygen in water, blood, and air samples. These techniques were conceived by what Krogh called “visual thinking” in a later autobiographical paper,^7^ and provided an unmatched precision that enabled the description of the influence of CO_2_ on blood oxygen binding^8^ as well as the realization that gas exchange across respiratory epithelia, including the human lung, occurs by diffusion alone.^9^

In 1902, the year before he defended his doctoral thesis, Krogh was invited to participate in a scientific expedition to Greenland, where he intended to study the respiratory gas exchange of the organisms of the Arctic sea using his newly developed equipment. However, soon after leaving Copenhagen on the sailing vessel *Peru*, Krogh shifted his attention to determining whether CO_2_ was indeed in equilibrium between fresh and sea waters and the atmosphere. In the introduction to the synthesizing paper from 1904, Krogh remarked: “*The results I attained with regard to the least mentioned question appeared to me to be so interesting that I devoted most of my time to it during my stay in Greenland, and I have since my return pursued it further*.”^4^

While at sea and while travelling up and down the west coast of Greenland, Krogh ascertained with very high precision that the CO_2_ tension in air was slightly higher than that of the waters and surmized that the atmospheric CO_2_ tension was on the rise. At that time, the explanations for a rise in atmospheric CO_2_ were far from clear, but Krogh reasoned that combustion of fossil fuels was the only credible explanation. He reasoned that “*The combustion of coal by man is an ever increasing factor that has in recent years reached very considerable magnitude—The worlds production of coal amounted in 1902 to 700 million tons giving by combustion 2.6×10^9^ ton of carbonic acid or rather more than 1/1000 of the quantity present in the atmosphere. In the geological insignificant period of 1000 years the per-centage of carbonic acid could therefore be doubled by this course alone, if other factors remained unchanged*.”^4^

Krogh's measurements provided some of the first conclusive evidence that atmospheric CO_2_ was actually on the rise. He was also amongst the first scientists to acknowledge that the ocean serves as a crucial CO_2_ buffer and he performed similar “back of the envelope,” but very reasonable, calculations showing that the oceans could absorb vast amounts of the CO_2_ produced. What Krogh did not predict, however, was the almost explosive rise in fossil fuel combustion that would ensue over the coming century, where we currently emit more than 35×10^9^ ton CO_2_ per year.
